# Distinct forebrain regions define a dichotomous astrocytic profile in multiple system atrophy

**DOI:** 10.1186/s40478-023-01699-3

**Published:** 2024-01-02

**Authors:** Y. Schneider, C. Gauer, M. Andert, A. Hoffmann, M. J. Riemenschneider, W. Krebs, N. Chalmers, C. Lötzsch, U. J. Naumann, W. Xiang, V. Rothhammer, R. Beckervordersandforth, J. C. M. Schlachetzki, J. Winkler

**Affiliations:** 1 Department of Molecular Neurology, University Hospital Erlangen, Friedrich-Alexander-University Erlangen-Nürnberg, Schwabachanlage 6, 91054 Erlangen, Germany; 2 UK Dementia Research Institute, The University of Edinburgh, Edinburgh, UK; 3 Institute of Biochemistry, Friedrich-Alexander-University Erlangen-Nürnberg, 91054 Erlangen, Germany; 4 Department of Neuropathology, Regensburg University Hospital, 93053 Regensburg, Germany; 5 Department of Neurology, University Hospital Erlangen, Friedrich-Alexander-University Erlangen-Nürnberg, 91054 Erlangen, Germany; 6 Core Unit Bioinformatics, Data Integration and Analysis (CUBiDA), University Hospital Erlangen, Friedrich-Alexander-Universität Erlangen-Nürnberg, 91054 Erlangen, Germany; 7 Keenan Research Centre for Biomedical Science, St Michael’s Hospital, Toronto, ON Canada; 8 Department of Immunology, The University of Toronto, Toronto, ON Canada; 9 Department of Cellular and Molecular Medicine, University of California-San Diego, La Jolla, CA 92093 USA

## Abstract

The growing recognition of a dichotomous role of astrocytes in neurodegenerative processes has heightened the need for unraveling distinct astrocytic subtypes in neurological disorders. In multiple system atrophy (MSA), a rare, rapidly progressing atypical Parkinsonian disease characterized by increased astrocyte reactivity. However the specific contribution of astrocyte subtypes to neuropathology remains elusive. Hence, we first set out to profile glial fibrillary acidic protein levels in astrocytes across the human *post mortem* motor cortex, putamen, and substantia nigra of MSA patients and observed an overall profound astrocytic response. Matching the *post mortem* human findings, a similar astrocytic phenotype was present in a transgenic MSA mouse model. Notably, MSA mice exhibited a decreased expression of the glutamate transporter 1 and glutamate aspartate transporter in the basal ganglia, but not the motor cortex. We developed an optimized astrocyte isolation protocol based on magnetic-activated cell sorting via ATPase Na+/K+ transporting subunit beta 2 and profiled the transcriptomic landscape of striatal and cortical astrocytes in transgenic MSA mice. The gene expression profile of astrocytes in the motor cortex displayed an anti-inflammatory signature with increased oligodendroglial and pro-myelinogenic expression pattern. In contrast, striatal astrocytes were defined by elevated pro-inflammatory transcripts accompanied by dysregulated genes involved in homeostatic functions for lipid and calcium metabolism. These findings provide new insights into a region-dependent, dichotomous astrocytic response—potentially beneficial in the cortex and harmful in the striatum—in MSA suggesting a differential role of astrocytes in MSA-related neurodegenerative processes.

**Supplementary Information:**

The online version contains supplementary material available at 10.1186/s40478-023-01699-3.

## Introduction

Astrocytes represent a highly heterogeneous glial population in the central nervous system (CNS) with diverse functions related to maintaining the integrity of the blood–brain barrier and the cerebral homeostasis of fluids, ions, pH, and neurotransmitters [1, 2]. Astrocytes contribute to protecting from glutamate-mediated excitotoxicity by removing glutamate from the synaptic cleft and preventing hyperstimulation of postsynaptic receptors [3, 4]. They also closely interact with neurons as well as blood vessels thereby regulating CNS metabolism [5] and, by storing and release of glycogen, transfer of glucose metabolites and generation of lactate, dynamically support neural functions [6–8]. Furthermore, astrocytes regulate lipid metabolism such as cholesterol efflux and oxidation of fatty acids [9, 10]. Besides regulating essential processes in the CNS, astrocytes have been described to closely interact with microglia and respond dynamically to cytokines secreted [11, 12]. This astro-microglial crosstalk was shown to result in conversion of astrocytes into a neurotoxic or neuroprotective state dependent on the stimuli (Fig. 2A) [11, 13].

For decades, expression of glial fibrillary acidic protein (GFAP), an astrocytic-specific marker in the brain, is used to define astrocytic identity and reactivity [1]. Expression of GFAP, however, varies among astrocyte subpopulations and is region-dependent; for example, grey matter regions demonstrate lower GFAP levels compared to white matter and hippocampus [14]. Therefore, using ubiquitously expressed astrocyte markers such as aldehyde dehydrogenase 1 family member L1 (ALDH1L1), aldolase C, or glutamine synthetase (GS) are important for a comprehensive characterization of astrocytic populations [1].

Rapid methodological developments resulted in the identification of transcriptional astrocytic expression profiles in physiological as well as pathological states [15–17]. These assignments led to the translation from structural phenotyping to region- and context-specific functions thereby identifying astrocytic subpopulations with a spectrum of potential effector functions ranging from neurotoxic properties with impaired autophagy, synapse elimination, and dopamine regulation [18, 19] to neuroprotective signatures promoting CNS recovery and repair by secreting transforming growth factor β (TGF-β) and brain derived neurotrophic factor (BDNF), angiopoietin-1 (ANG1), and expression of connexin-43 (CX43) [20–22].

Multiple system atrophy (MSA), a rare sporadic oligodendroglial α-synucleinopathy, is a rapidly progressing neurodegenerative disease with an average survival rate of 6–9 years [23–25]. Clinically, MSA is subdivided into a parkinsonian (MSA-P) and cerebellar phenotype (MSA-C) accompanied by an early and severe autonomic failure [26]. In MSA-P, the dorsolateral putamen, caudate nucleus, and substantia nigra display pronounced neuronal loss whereas cortical areas such as the precentral gyrus are less affected [27]. Reactive astrogliosis has been shown in the putamen of MSA patients demonstrating an inverse correlation between reactive astrocytes and the distance to glial cytoplasmic inclusions [28, 29].

To shed more light into astrocytic reactivity in MSA, we first examined *post mortem* prototypical MSA-P brain regions to determine reactive astrogliosis in the cortex, the striatum, and the substantia nigra. Next, to test if astrocyte reactivity observed in *post mortem* tissue is recapitulated in a mouse model of MSA, we took advantage of the MBP29-hα-syn mouse model.

In 2005, Shults and colleagues generated a mouse model overexpressing human α-synuclein (hα-syn) in oligodendrocytes under the control of a myelin-basic-protein (MBP) specific promotor (MBP29-hα-syn) [30]. These mice start to develop progressive motor deficits at the age of 10 weeks, prematurely die between 20 and 30 weeks of age, and show a severe neuronal loss accompanied by a widespread demyelination and region-specific microgliosis matching important functional and neuropathological features of MSA [31, 32]. Due to the important role of astrocytes to alter neurodegenerative and inflammatory processes [33–35] there is an urgent need to comprehensively characterize astrocytic subpopulations in MSA and its respective model for identifying potential targets suitable for future interventional strategies. After confirming a similar astrocytic phenotype in MBP29-hα-syn mice, we made use of an optimized state-of-the-art isolation protocol using magnetic activated cell sorting (MACS) approach to decipher the transcriptional profile of astrocytes in CNS regions differentially affected by the oligodendroglial synucleinopathy.

## Material and methods

### Human *post mortem* brain tissue

*Post mortem* cortex, putamen and substantia nigra of MSA-P patients and age- and sex-matched controls (each n = 4, Table 1) were obtained from the Netherlands Brain Bank (NBB), Netherlands Institute for Neuroscience, Amsterdam (open access: http://www.brainbank.nl). MSA cases were clinically diagnosed and neuropathologically confirmed as MSA-P according to the second consensus criteria (Table 1; [36]). Paraffin-embedded tissue of the cortex, the striatum, and the substantia nigra were sectioned at a thickness of 5 µm.

**Table 1:** Overview of MSA-P patients with clinical characteristics and controls

Diagnosis	Gender	Age (year)	*Post mortem* delay (min)	Disease duration (month)	Brain weight (g)
MSA-P	F	66	485	88	1005
MSA-P	M	67	370	77	1376
MSA-P	F	67	435	97	1244
MSA-P	F	59	400	54	1102
Control	F	77	340	–	1111
Control	F	60	450	–	1240
Control	M	51	465	–	1450
Control	M	55	435	–	1393

### Animals

Transgenic MBP29-hα-syn mice overexpressing human α-syn under the control of the murine MBP promoter and corresponding non-transgenic littermates (NTG) serving as controls were anesthetized and sacrificed at an age of 4 weeks. For immunofluorescence staining, animals were perfused using 4% paraformaldehyde (PFA) and 0.9% sodium chloride solution according to the European and National Institute of Health guidelines for humane treatment. Afterwards, brains were transferred into 4% PFA for 2–4 h and subsequently stored in 30% sucrose solution. Whole forebrains were sagittally sectioned at a thickness of 40 µm and stored in cryoprotection solution (25% 0.2 mol/L phosphate buffer) at − 20 °C until further staining. For expression analysis of proteins and mRNA, by Western blot (WB) and quantitative PCR (qPCR), respectively, mice were perfused using phosphate-buffered saline (PBS). After the removal of the brain, the cortices and striata were subsequently micro-dissected, snap-frozen in liquid nitrogen, and stored at −80 °C until homogenization. For the isolation of astrocytes and oligodendrocytes, mice were sacrificed, brains were removed, dissected as described above and stored at 4 °C in Dulbecco’s Phosphate Buffered Saline containing MgCl_2_ and CaCl_2_ (D-PBS) until further processing for MACS.

### Immunohistochemistry of human *post mortem* tissue

Immunohistochemistry on formalin-fixed and paraffin-embedded tissue was performed following a previously published protocol [31]. In brief, sections at a thickness of 5 µm were deparaffinized. After microwave antigen retrieval using citrate buffer, sections were stained using the REAL EnVision Detection System peroxidase/DAB+, Rabbit/Mouse (Dako, # K5007) according to the manufacturer’s protocol. The kit uses 3,3′-diaminobenzidine tetrahydrochloride (DAB) as a chromogen. Finally, sections were counterstained using hematoxylin. Primary antibodies and staining conditions were as follows: antigen retrieval using citrate buffer (0.1 M) for 30 min followed by anti-GFAP (Agilent Cat# M0761, RRID:AB_2109952, 1:100) incubation overnight. As positive control, an intracerebral metastasis of a breast carcinoma with an adjacent reactive border zone was used. Analyzed areas of each region is given in Additional file 1: Table S2.

### Immunofluorescence staining of human *post mortem* brain tissue

Brain sections were deparaffinized by heating at 60 °C for 1 h followed by incubation in xylene and re-hydrated with a descending ethanol series (100%/95%/70%). Antigen retrieval was performed using Citrate-EDTA buffer (10 mM Citric acid, 2 mM EDTA, 0.05 Tween-20, pH 6.2) at 95 °C for 15 min. Afterwards, sections were permeabilized with 1% Triton-X100 in PBS for 30 min. Sections were incubated with primary antibodies at 4 °C for 72 h: glial fibrillary acidic protein GFAP (Agilent Cat# GA524, RRID:AB_2811722, 1:500) and excitatory amino acid transporter 2 (Novus Cat# NBP1-20136, RRID:AB_2190752, 1:500). Primary antibodies were followed by the incubation of secondary antibodies: donkey anti-rabbit Alexa Fluor 488 (Molecular Probes Cat# A-21206, RRID:AB_2535792, 1:500) and donkey anti-mouse Cy3 (Millipore Cat# AP192C, RRID:AB_92642, 1:500) at 4 °C overnight. Nuclei were counterstained using 4′,6-diamidino-2-phenylindole (DAPI). Imaging was performed using the fluorescence Observer microscope (Carl Zeiss Microscopy GmbH, Jena, Germany).

### Immunofluorescence staining of murine forebrain

For immunofluorescence staining, brain sections of six animals per genotype were utilized. Remaining cryoprotection solution was removed by rinsing with PBS. For improvement of antibody penetration, sections were incubated in 0.3 M glycine, 0.2% Triton-X100, and 10% DMSO in dH_2_O at room temperature (RT) for 1 h. Furthermore, unspecific antibody binding was blocked using 0.3% donkey serum, 0.2% Triton-X100 in PBS at 37 °C for 1 h. For astrocyte labeling, primary antibodies were incubated at 4 °C for 72 h: GFAP (Abcam Cat# ab4674, RRID:AB_304558, 1: 1.000), ALDH1L1 (Abcam Cat# ab87117, RRID:AB_10712968, 1:500), and S100β, (Abcam Cat# ab52642, RRID:AB_882426, 1:200). Forebrain sections were rinsed using PBS and subsequently incubated with secondary antibodies at RT for 1 h: anti-chicken Alexa 488 (Jackson ImmunoResearch Labs Cat# 703-545-155, RRID:AB_2340375, 1:500) and anti-rabbit Alexa 647 (Jackson ImmunoResearch Labs Cat# 711-605-152, RRID:AB_2492288, 1:500). Secondary antibody was rinsed and nuclei were counterstained using DAPI. Imaging was performed using the Axio Observer fluorescence microscope (Carl Zeiss Microscopy GmbH, Jena, Germany). Images were processed and analyzed applying ImageJ and Fiji software [37, 38].

### Isolation of murine astrocytes and oligodendrocytes

Adult astrocytes and oligodendrocytes were isolated using MACS. Generation of single cell suspensions, myelin and red blood cell removal was performed using the Neural Tissue Dissociation Kit (P) (130-092-628, Miltenyi Biotec). For isolation of cortical and striatal glial cells, olfactory bulbs and hindbrain were removed and the respective regions of five animals were pooled. After adding papain to samples in C-tubes (130-093-237, Miltenyi Biotec), homogenization of the samples was performed with program 37C_ABDK_01 of the gentleMACS™ Octo Dissociator with Heaters (130-096-427, Miltenyi Biotec). To obtain a sufficient amount of RNA, five animals of mixed sex per genotype were pooled for each sample used for subsequent bulk RNA sequencing (for 3n in total: 15 animals/genotype per region). After removal of debris and red blood cells, Fc-receptors (FcR) were blocked by incubation of single cell suspension using FcR Blocking Reagent (mouse, 130-092-575, Miltenyi). Astrocytes were magnetically labelled using ACSA-2 MicroBeads (130-097-678, Miltenyi Biotec) according to the manufacturer’s protocol. O4^+^ oligodendrocytes were pre-labeled with anti-O4 MicroBeads (human, mouse, rat, 130-094-543, Miltenyi Biotec). The generated cell suspensions were passed through MS columns twice (130-097-678, Miltenyi Biotec) by performing two runs applying a magnetic field by the OctoMACS™ manual separator (130-042-109, Miltenyi Biotec) modified from previously published protocols [39]. ACSA2^+^ astrocytes were flushed into Eppendorf Tubes^®^ in AstroMacs Separation Buffer. Cell pellets were collected and consecutively lysed in Buffer RLT Plus (1053393, Qiagen) for RNA isolation, resuspended in 2% bovine serum albumin in D-PBS (FC buffer) for flow cytometry, or snap-frozen in liquid nitrogen, and stored at − 80 °C until further use.

### Flow cytometry of murine astrocytes and oligodendrocytes

Cell suspensions were centrifuged in FC buffer. Subsequently, cell pellets were resuspended in FC buffer containing fluorophore-conjugated antibodies: the astroglia recognizing antibody ACSA2, APC (Miltenyi Biotec Cat# 130-123-284, RRID:AB_2811488, 1:75) and the oligodendroglia recognizing antibody O4 Phycoerythrin (R and D Systems Cat# FAB1326P, RRID:AB_664169, 1:75). For proper labeling, cells were incubated with antibodies at 4 °C for 15 min. Pellets were resuspended in cold FC buffer and dead cells were stained using DAPI. ACSA2^+^ and O4^+^ cells were subsequently analyzed using the BD LSRFortessa™ Cell Analyzer (BD Biosciences GmbH). Quantification of ACSA2^+^ and O4^+^ populations were calculated using FlowJo™ Software (BD Biosciences GmbH).

### RNAscope

Prior to RNAscope analysis, forebrains were embedded in Tissue Tek O.C.T Compound (SA-4583, Sakura), snap-frozen and sectioned at a thickness of 14 µm. For RNAscope, sections were heated at 60 °C for 30 min and post-fixed in 4% PFA at 4 °C. Sections were dehydrated using an ascending alcohol series (50/70/100% ethanol) and air-dried afterwards. Tissue was further dehydrated with hydrogen peroxide. Antigen-retrieval was performed using the RNA–Protein Co-detection Ancillary Kit (323180, ACD). Primary antibodies were diluted in Co-detection Antibody Diluent (323180, ACD) and incubated at 4 °C overnight: GFAP (Abcam Cat# ab4674, RRID:AB_304558, 1: 1.000) and glutamine synthetase (GS) (Santa Cruz Biotechnology Cat# sc-74430, RRID:AB_1127501, 1:500). Sections were fixed using 2% PFA after primary antibody incubation. Additionally, sections were treated with Protease III according to manufacturer’s protocol. Afterwards, probes were hybridized at 40 °C for 2 h: RNAscope Probe-Mm-Myrf-C2 (524061-C2, Biotecne), and RNAscope Probe-Mm-Sox10 (435931, Biotecne). Signal amplifiers (AMP1 and 2) and fluorophores (TSA Vivid Fluorophore 520/650, 7527/7523, TOCRIS) were linked to the probes at 40 °C for 30 min, respectively. Fluorophore-conjugated secondary antibodies were diluted in Co-detection Antibody Diluent and linked to primary antibodies at RT for 30 min. Nuclei were counterstained using DAPI and sections were mounted in Aqua-Poly/Mount.

### Western blotting (WB)

For WB analysis, five animals per genotype were processed. Total protein amount was calculated to 15 µg in radioimmunoprecipitation assay (RIPA) buffer: 50 mM Tris–HCl (pH 8.0, 150 mM NaCl, 2 mM EDTA, 1% v/v Nonidet P-40 (Roche), 0.5% v/v Na-deoxycholate (Roth), 0.1% w/v SDS (Applichem)) complemented with protease inhibitor (11697498001, Roche), and phosphatase inhibitor (4906837001, Roche) for SDS-PAGE on 4–12% Bis–Tris gels (NP0322BOX, Invitrogen). Protein was blotted on polyvinylidene difluoride membranes for fluorescence applications (PVDF-FL, IPFL 00010, Millipore). Unspecific antibody binding was blocked using 1% bovine serum albumin followed by incubation at 4 °C overnight with the following primary antibodies: GFAP (Abcam Cat# ab4674, RRID:AB_304558, 1: 1.000), vimentin (VIM) (Abcam Cat# ab20346, RRID:AB_445527, 1:1.000), GLT-1 (Millipore Cat# AB1783, RRID:AB_90949, 1:500), GLAST (Abcam Cat# ab41751, RRID:AB_955879, 1:500), GS (Santa Cruz Biotechnology Cat# sc-74430, RRID:AB_1127501, 1:500), aquaporin-4 (AQP-4) (Sigma-Aldrich Cat# A5971, RRID:AB_258270, 1:500), and growth associated protein 43 (GAP-43) (Millipore Cat# MAB347, RRID:AB_94881, 1:1.000). Primary antibodies were rinsed and followed by secondary antibodies at RT for 1 h: anti-mouse Alexa Fluor 488 (Molecular Probes Cat# A-21202, RRID:AB_141607, 1:1.000), anti-rabbit Alexa Fluor 488 (Molecular Probes Cat# A-21206, RRID:AB_2535792, 1:1.000), anti-chicken Alexa Fluor 488 (Jackson ImmunoResearch Labs Cat# 703-545-155, RRID:AB_2340375, 1:500), anti-rabbit Alexa Fluor 647 (Jackson ImmunoResearch Labs Cat# 711-605-152, RRID:AB_2492288, 1:500), and anti-mouse Alexa Fluor 568 (Thermo Fisher Scientific Cat# A10037, RRID:AB_2534013, 1:1.000).

### RNA isolation

RNA was isolated using the RNeasy Plus Micro Kit (74034, Qiagen). Cells were lysed in RLT Plus Buffer (+ β-mercaptoethanol, 1:100). Homogenized lysate was transferred to gDNA eliminator spin. RNA was transferred to a RNeasy spin column and eluted into new tubes according to manufacturer’s protocol. Total RNA concentration was measured using the NanoDrop^®^ ND-1000 (Thermo Scientific) and stored at − 80 °C until bulk RNA sequencing or quantitative real-time PCR.

### Quantitative real-time PCR (qRT-PCR)

Coding DNA (cDNA) was generated using GoScript™ Reverse Transcription System according to manufacturer’s protocol. For the analysis of gene expression, the SSoFast™ EvaGreen^®^ Supermix was mixed with respective primers (Additional file 1: Table S1) and amplified using the Light Cycler 480 system. For the analysis, gene expression levels were normalized to housekeeper genes Tyrosine 3-Monooxygenase (*Ywhaz*) and Glucuronidase Beta (*Gusb*). For qPCR, 4 animals per genotype were analyzed.

### Bulk RNA sequencing

Samples were submitted to Genewiz GmbH (Leipzig, Germany) to prepare a standard and ultra-low input RNA-seq [40] library prior sequencing using an Illumina HiSeq platform. Reads were delivered as fastq files. Subsequent pre-processing and quality control of fastq files was performed by the Core Unit of Bioinformatics, Data Integration and—Analysis (CuBiDa) of the University Hospital Erlangen, Erlangen, Germany.

### Bioinformatic analysis

In the first step of the bioinformatics data analysis of bulk RNA-seq data, the quality of the raw paired-end sequencing data sets (fastq) was assessed with the tool FastQC v0.11.9 [41]. Samples that passed quality assessments were further optimized by a trimming step with the tool Trimmomatic v0.39 [42]. Resulting samples were mapped with STAR aligner v2.7.9a [43] utilizing the mouse reference genome (Mus_musculus.GRCm39) from Ensembl release 104. The aligned sequences were converted to raw read counts per exon/gene with the tool featureCounts which is included in the Rsubread software package v2.14.2 [44]. Gene symbol annotation was performed using the AnnotationDbi package [45]. Modelling, normalization, and differential gene expression analysis was performed using the DESeq2 package [46] in combination with the R Version 4.1.2. Log2 fold-changes were set to > 1 and adj. p-values to 0.05. Differential expressed genes were pre-ranked by stats and gene set enrichment performed using the fgsea package. Results were visualized using the ggplot2 package. To determine cell lineages, the enricher-function of clusterProfiler package [47] was applied on the 50 most highly expressed transcripts. For assessment of cellular identity, the gene list was enriched on a single-cell RNA sequencing based mouse cell-marker dataset: CellMarker 2.0 [48]. *P* value threshold was set to 0.05 and *p* value adjustment was performed using Benjamini–Hochberg (BH) procedure. Results were visualized using ggplot2 package.

### Statistical analyses

Data were analyzed and visualized using GraphPad Prism 9.5.0 and CorelDraw X6 software. Shapiro–Wilk test was performed for determination of normal distribution of expression levels and cell counts. Welch’s t-test was conducted for determination of statistical significance. If no Gaussian distribution was determined, statistical analysis was performed using Mann–Whitney–U test. Unless otherwise stated, all graphs represent mean values and standard deviation. *P* values were defined as statistically significant at *p* < 0.05.

## Results

### Severe astrocytic response in the cortex, the putamen, and the substantia nigra of MSA-P patients

**Fig. 1:**
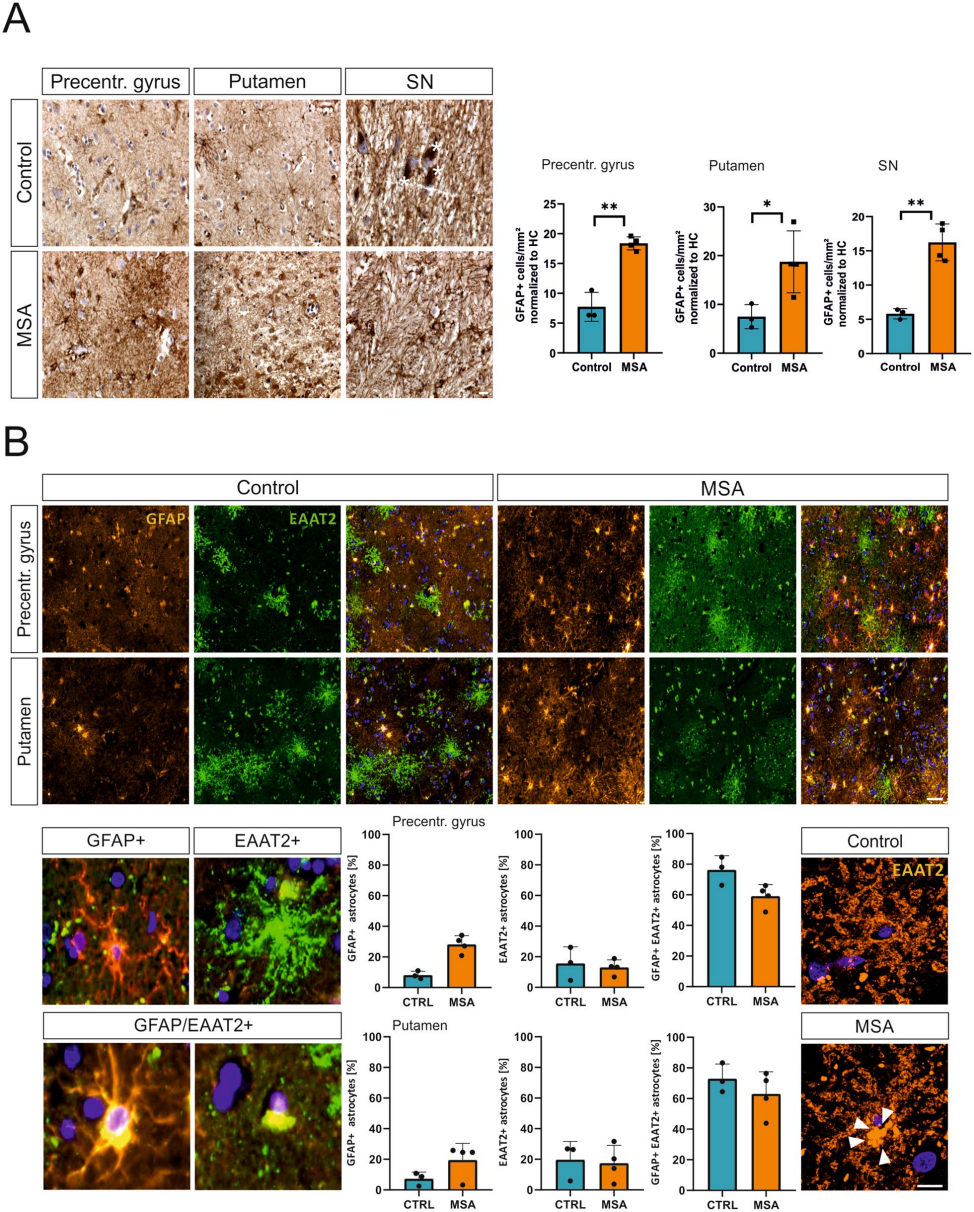
Astrogliosis in *post mortem* brain tissue of MSA patients. **A** Representative image of DAB staining of GFAP^+^ astrocytes in precentral gyrus, putamen, and substantia nigra of MSA-P patient (female, 67) and control individual (female, 60) and quantification of GFAP^+^ cells/mm^2^ (4 MSA patients vs. 4 Controls). SN was identified by presence of neuromelanin-containing neurons (white asterisks). Welch’s t-test was used for statistical analysis. Scale bar = 20 µm. All three regions display elevated numbers of GFAP^+^ astrocytes in cortex (*p* = 0.002), putamen (*p* = 0.0003), and substantia nigra (*p* < 0.0001) of MSA patients. CTRL = Control, MSA = multiple system atrophy, SN = substantia nigra. (**B**, Upper panel) Immunofluorescence staining of four MSA-P patients and three controls. For visualization of astrocytes GFAP was used as a marker (orange). To analyze expression of glutamate reuptake transporter tissue was stained for EAAT2 (green). Scale bar = 50 µm. (**B**, Lower panel) Overview of single cells expressing GFAP, EAAT2, and GFAP/EAAT2 (yellow, lower panel). Astrocytic GFAP/EAAT2 expression is decreased in the precentral gyrus (*p* = 0.0571). Moreover, a re-distribution towards the cytoplasm of EAAT2 is observed in astrocytes of MSA patients (Lower right panel)

The upregulation of GFAP is indicative for the response of astrocytes to physiological or pathological stimuli [1]. Based on a previous study by Hoffmann and colleagues [31] observing severe microgliosis in putaminal white matter of MSA patients and MBP29-hα-syn mice, we assessed GFAP reactivity exclusively in grey matter of the putamen. GFAP^+^ branched reactive astrocytes were increased in the motor cortex, the putamen, and the substantia nigra of MSA-P patients compared to age- and sex-matched controls (Fig. 1A, B). The proportional highest increase of GFAP^+^ reactive astrocytes was observed in the substantia nigra (2.8-fold; *p* < 0.0001) followed by the putamen (grey matter, 2.5-fold; *p* = 0.0003) and the motor cortex (precentral gyrus, 2.4-fold; *p* = 0.002).

Astrocytes are neural cells predominantly expressing high-affinity glutamate transporters involved in regulating glutamate levels within the synaptic cleft. Therefore, we asked whether astrocytic expression levels of the excitatory amino acid transporter-2 (EAAT2; mouse homologue GLT-1) are altered in the cortex and the putamen of MSA-P patients (Fig. 1B, upper panel). Interestingly, we observed a decreased number of GFAP^+^/EAAT2^+^ astrocytes in the precentral gyrus of MSA patients (*p* = 0.0571). However, we did not observe changes in GFAP^+^/EAAT2^+^ in the putamen of controls and MSA-patients (*p* = 0.6286, lower panel). Although no differences in the expression levels were observed, EAAT2^+^ astrocytes in MSA-P patients display accumulations of EAAT2 towards the nucleus indicating changes in distribution of EAAT2 in MSA. Due to the small sample size the quantification of the nuclear and diffuse distribution pattern did not reach statistical significance, but hints toward the nuclear distribution in the putamen of MSA-P patients (Additional file 6: Fig. S5). These findings of changed expression pattern of EAAT2 in the cortex and the putamen of MSA-P patients suggests impairments in essential functions of astrocytes caused by MSA-related pathology.

### Pronounced astrocytic response in the cortex, the striatum, and the substantia nigra of MBP29-hα-syn mice

**Fig. 2:**
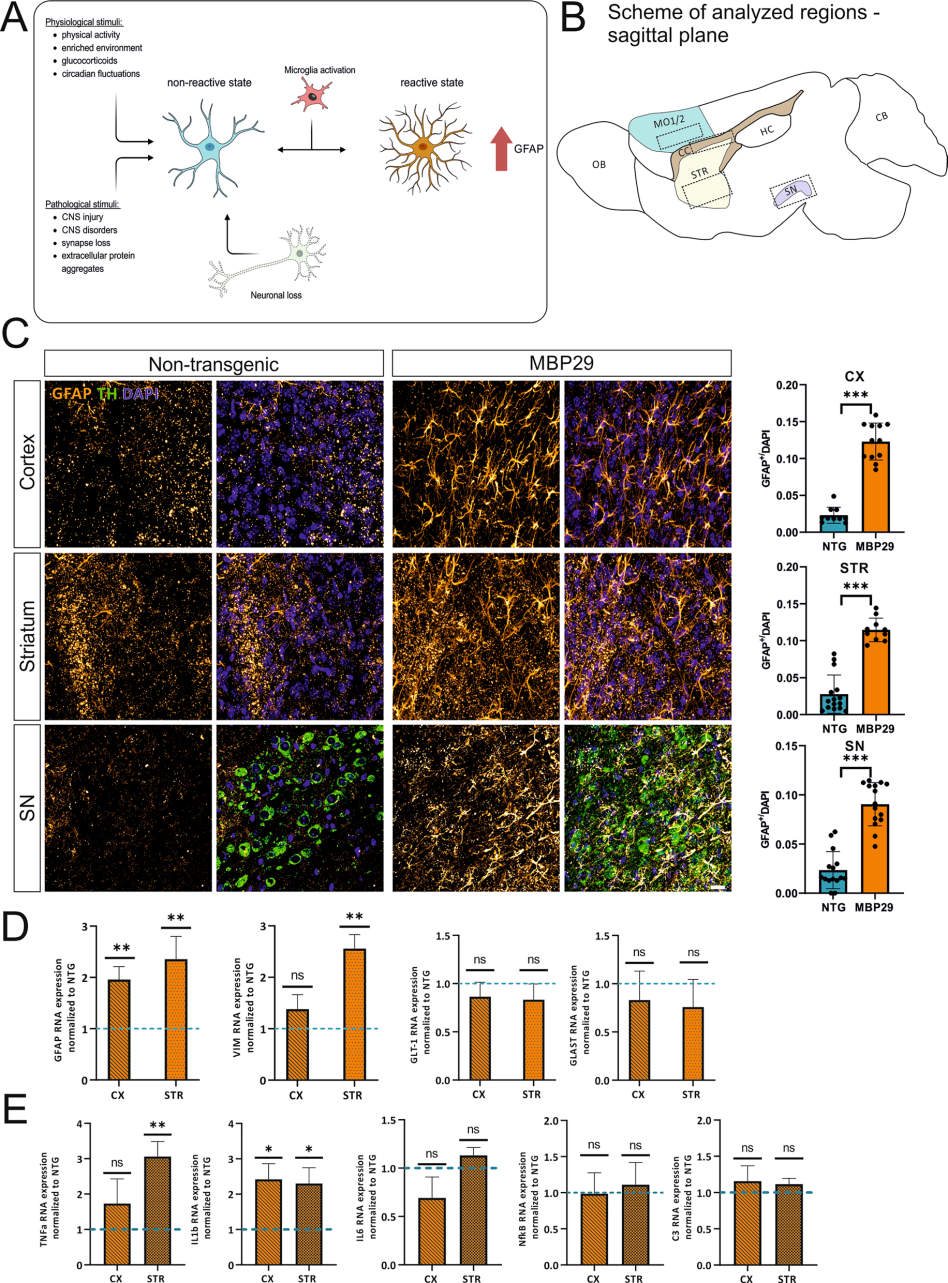
Astrogliosis and differential inflammatory signature in brain regions of MBP29-hα-syn mice. **A** Overview of stimuli inducing upregulation of astrocytic GFAP expression. Astrocytes respond to either pathological or physiological stimuli. Alternatively, microglial subtypes or neuronal loss may result in increased astrocytic GFAP expression. Consequently, GFAP^+^ upregulated astrocytes show complex and multibranched processes [1]. Created with BioRender.com. **B** CNS regions analyzed of MBP29-hα-syn mice (sagittal plane). MO1/2 = Motor cortex 1/2, CC = corpus callosum, STR = striatum, SN = substantia nigra pars compacta, OB = olfactory bulb, HC = hippocampus, CB = cerebellum. **C** Characterization of GFAP^+^ expressing astrocytes in the cortex, the striatum, and the substantia nigra of MBP29-hα-syn mice and non-transgenic controls (NTGs). Dopaminergic neurons of the substantia nigra were visualized by tyrosine hydroxylase (TH, green; lower panel). The number of GFAP^+^ astrocytes was dramatically increased in the cortex (*p* < 0.001), the striatum (*p* < 0.001), and the substantia nigra (*p* < 0.001) of MBP29-hα-syn mice compared to NTGs. The numbers of GFAP^+^ astrocytes (n = 6/group/region) are shown as mean ± SD (right column); *** *p* < 0.001, Scale bar = 20 µm. **D**, **E** qPCR analysis of cortical and striatal microdissected tissues of MBP29-hα-syn mice and non-transgenic controls (NTGs, n = 4 animals per genotype): GFAP, VIM, and the glutamate reuptake transporters (glutamate transporter-1 (*Glt-1*) and the glutamate-aspartate transporter 1 (*Glast*)) (**D**) as well as pro-inflammatory cytokines such as tumor-necrosis factor α (*Tnfa*), interleukin-1b (*Il1b*), interleukin-6 (*Il6*), nuclear factor kappa B (*Nfkb*), and complement component 3 (C3) (**E**). Striatal expression levels of GFAP (*p* = 0.006) and VIM (*p* = 0.002) are significantly increased in MBP29-hα-syn mice, however GFAP is solely elevated in the cortex of MBP29-hα-syn mice (*p* = 0.001). RNA expression of GLT-1 and GLAST are not altered in both regions (**D**). *Tnfa* expression is elevated by threefold in the striatum of MBP29-hα-syn mice only (*p* = 0.003). In contrast, *Il1b* levels are similarly increased in both regions, the cortex (*p* = 0.0312) and the striatum (*p* = 0.0367) of transgenic mice. *Il6* , *Nfkb*, and *C3* expression is not altered compared to NTGs (**E**). Quantification (n = 4 animals/group) is shown as mean ± SD. *p < 0.05, **p < 0.01, and ***p < 0.005

Based on the *post mortem* findings in MSA-P patients, we characterized the astrocytic response in MBP29-hα-syn mice at 4 weeks of age, an early (pre-motor) stage of this model [30]. First, we confirmed the presence of alpha-synuclein (aSyn) pathology and myelination deficits in MBP29-hα-syn mice at 4 weeks of age (Additional file 5: Fig. S4). Furthermore, we quantified the number of GFAP^+^ astrocytes in the cortex (pure grey matter) and the clinically most affected regions of the MBP29-hα-syn mice, the striatum (mixed grey and white matter) as well as the substantia nigra (pars compacta, grey matter; Fig. 2B). A significant astrocytic response was present in the cortex, the striatum, and the substantia nigra of MBP29-hα-syn mice compared to NTGs (Fig. 2C, left panel). The proportional highest increase of GFAP^+^ astrocytes was observed in the striatum (fourfold; *p* < 0.001) followed by the substantia nigra (3.5-fold; *p* < 0.001) and the motor cortex (2.5-fold; *p* < 0.001; Fig. 2C, right panel).

ALDH1L1 represents a pan-astrocyte marker labeling astrocyte independently of the expression of GFAP [15]. To exclude that the observed increase in GFAP expression is solely based on the overall increase of astrocytes, we calculated the ratio of GFAP^+^ and ALDH1L1^+^ for both genotypes respectively (Additional file 3: Fig. S2). By analyzing the number of astrocytes expressing ALDH1L1 in these regions, we observed an overall elevated number of ALDH1L1^+^ astrocytes only in the cortex of MBP29-hα-syn mice compared to NTGs (> 1.5-fold) (Additional file 3: Fig. S2). These results confirm that the increased number of GFAP^+^ cells is not predominantly due to an increased recruitment of astrocytes or new-born astrocytes, but rather GFAP expression is triggered by the region-specific microenvironment.

To confirm the increased GFAP protein levels in astrocytes in MBP29-hα-syn, we performed qPCR analyses of cortical and striatal tissue homogenates (Fig. 2D). GFAP mRNA was increased in the cortex and striatum by 2-fold (*p* = 0.001) and 2.5-fold (*p* = 0.006), respectively. In contrast, mRNA levels of VIM, an important intermediate filament of astrocytes, was significantly increased in the striatum only (2.5-fold, *p* = 0.002). Furthermore, we focused on the gene expression levels of the glutamate reuptake transporters GLT-1 and GLAST to assess altered expression of transcripts essential for astrocytic functionality. We did not detect differences in RNA levels for GLAST (cortex: 0.8-fold, *p* = 0.36; striatum: 0.75-fold, *p* = 0.2) and GLT-1 (cortex: 0.85-fold, *p* = 0.2; striatum: 0.83-fold, *p* = 0.13).

As inflammatory processes induce an increased response of astrocytes in the CNS by up-regulation of pro-inflammatory transcriptional pathways, we searched for altered cytokine expression levels in MBP29-hα-syn mice. We detected a strong significant transcriptional increase of the tumor-necrosis factor a (*Tnfa*) in the striatum (threefold, *p* = 0.0034) and, albeit to a lesser degree, in the cortex (1.7-fold, *p* = 0.26). On the other hand, cortical and striatal interleukin-1-b (*Il1b*) gene expression levels were significantly elevated (cortex: 2.4-fold, *p* = 0.031; striatum: 2.3-fold, *p* = 0.034; Fig. 2E). Levels of other inflammation-associated molecules such as interleukin-6 (*Il6*) (cortex: 0.69-fold, *p* = 0.34; striatum: 1.1-fold, *p* = 0.3), nuclear factor kappa-light-chain-enhancer of activated b-cells (*Nfkb*) (cortex: 0.98-fold, *p* = 0.96, striatum: 1.1-fold, *p* = 0.58), and *C3* (cortex: 1.2-fold, *p* = 0.32; striatum: 1.2-fold, *p* = 0.69) were not different between both groups (Fig. 2E). These findings suggest that proinflammatory processes may take place in the striatum (elevated TNFa and IL-1b), but demonstrating a distinct signature in the cortex (IL-1b) of MBP29-hα-syn mice.

### Striatal astrocytes display a proinflammatory phenotype accompanied by transcriptional downregulation of important homeostatic functions in MBP29-hα-syn mice

**Fig. 3:**
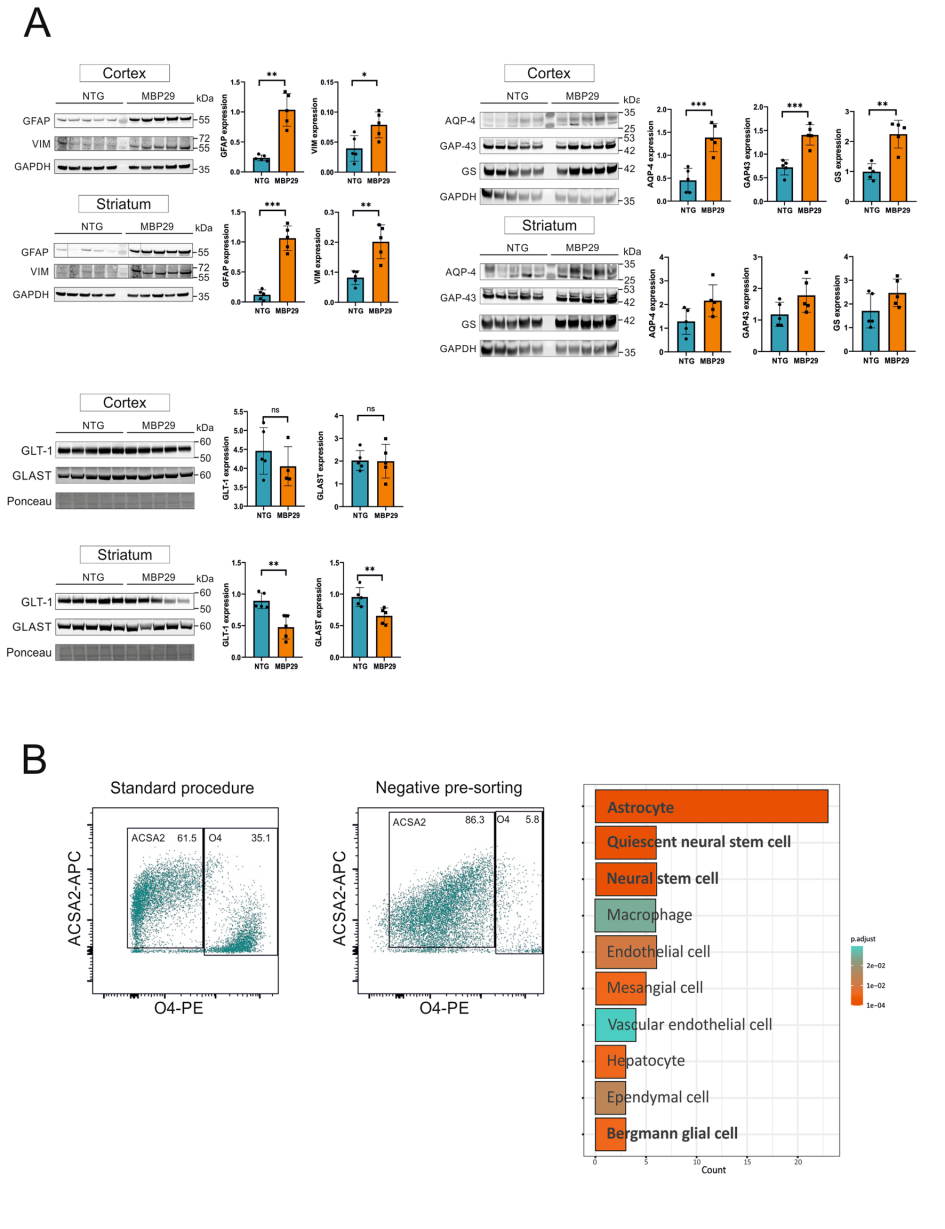
**A** Response- and homeostasis-associated protein levels of astrocyctes derived from MBP29-hα-syn and non-transgenic littermates. Upper left panel: Cortical and striatal levels of GFAP were increased in MBP29-hα-syn mice by ~ 4-(*p* < 0.001) and ~ 8-fold (*p* < 0.001), respectively. Similarly, there was an increased cortical and striatal level for VIM in MBP29-hα-syn mice by ~ 2-(*p* = 0.007) and ~ 2.5-fold (*p* < 0.001), respectively. Quantification of WBs (n = 5/ each group/ region) is shown as mean ± SD. Glyceraldehyde-3-phosphate dehydrogenase (GAPDH) served as control. kDa = kilo Dalton. *p < 0.05, **p < 0.01, ***p < 0.005. Upper right panel: AQP-4 levels are highly increased by ~ 3-fold in the cortex of MBP29-hα-syn mice (*p* < 0.001). Note the much lower, but not significant increase of AQP-4 levels in the striatum of MBP29-hα-syn mice (*p* = 0.054). A proportional and similar upregulation is present for growth associated protein-43 (GAP-43) levels showing a ~ 2-fold (*p* < 0.001) and ~ 1.5-fold (*p* = 0.074) increase in the cortex and the striatum of MBP29-hα-syn mice. A similar pattern is observed for GS by a ~ 2.5-fold increase (*p* < 0.001) in the cortex of MBP29-hα-syn mice, however without changing striatal levels. Quantification of WBs (n = 5 animals/group/region) is shown as mean ± SD. Glyceraldehyde-3-phosphate dehydrogenase (GAPDH) served as control. kDa = kilo Dalton. *p < 0.05, **p < 0–01, ***p < 0.005. Lower left panel: Protein levels of glutamate reuptake transporters glutamate transporter-1 (GLT-1) and glutamate-aspartate transporter 1 (GLAST) are not altered in the cortex of MBP29-hα-syn mice. In contrast, both glutamate reuptake transporters were reduced by ~ 2-fold (GLT-1, *p* = 0.001; GLAST, *p* = 0.005). Quantification of WBs (n = 5 animals/group/region) is shown as mean ± SD. Due to overlapping WB signals of GLT-1, GLAST, and GAPDH, whole protein Ponceau-S staining served as control. *p < 0.05, **p < 0.01, ***p < 0.005. **B** Isolation of astrocytes via MACS and cellular phenotyping. Flow cytometry analysis of magnetic activated cell sorting (MACS) of striatal and cortical astrocytes using the astrocyte cell surface antigen-2 (ACSA2) without (standard procedure; ACSA-2^+^) or with a preceding, negative sorting step for the oligodendrocyte marker 4 (negative pre-sorting, O4^−^, ACSA2^+^). Without removing O4^+^-cells, the purity of ACSA2^+^-cells amounts to 61.5% (left panel); a substantial O4^+^ cell population (35.1%) was still present. By adding the O4 presorting step, a highly improved purity of ACSA2^+^-cells (86.3%) was achieved; much less O4^+^-cells (5.8%) were detected (middle panel). Y-axis: logarithmic fluorescence of allophycocyanin (APC)-signal. X-axis: logarithmic fluorescence of phycoerythrin (PE)-signal. Each cell population analyzed is marked by a black rectangle, the proportion of ACSA2^+^ and O4^+^-cells is given in % of the entire cell population. Enrichment of the 50 most highly expressed genes in the isolated cell population using clusterProfiler. Enrichment analysis revealed the most significant enriched genes associated with astrocytes (right panel). Neural stem cells show a similar gene expression pattern

To further evaluate the presence of astrocyte markers that relate to different astrocyte subsets in the cortex and the striatum, we determined GFAP and VIM levels in both regions by western blotting (Fig. 3A, upper left panel). Indeed, striatal GFAP levels were significantly elevated by 8.2-fold in MBP29-hα-syn mice (*p* < 0.001) and to a lower degree in the cortex (4.4-fold; *p* < 0.001). The protein levels of VIM were similarly increased in both regions of the MBP29-hα-syn mice, but at smaller magnitude compared to GFAP (cortex: twofold, *p* = 0.0065; striatum: 2.5-fold, *p* < 0.001).

To determine region-specificity of astrocytic subpopulations, the protein levels of astrocyte-specific proteins (functions, see Table 2, Fig. 3A, upper right panel) such as AQP-4, GAP-43, and GS, as well as both glutamate reuptake transporters GLT-1 and GLAST (Fig. 3A, lower left panel) were quantified in the cortex and the striatum of MBP29-hα-syn mice and NTGs.

**Table 2:** Overview of astrocytic proteins involved in CNS homeostasis and their associated functions

Protein	Function
Aquaporin-4 (AQP4)	CNS water homeostasis
Glial fibrillary acidic protein (GFAP)	Intermediate filament of mature astrocytes
Growth-associated protein 43 (GAP-43)	Regenerative response, microglia activation
Glutamate transporter 1 (GLT-1)	Synaptic L-glutamate clearance
Glutamate-aspartate transporter 1 (GLAST)	Termination excitatory neurotransmission
Glutamine synthetase (GS)	Glutamine synthesis from glutamate
Vimentin (VIM)	Intermediate filament for cytoskeletal organization

Surprisingly, we observed elevated levels of astrocyte-associated proteins in both regions, albeit to a varying degree (Fig. 3A). The increased protein levels of AQP-4 (cortex: threefold, *p* < 0.001; striatum: 1.7-fold, *p* = 0.0135), GAP-43 (cortex: twofold, *p* < 0.001; striatum: 1.5-fold, *p* = 0.02), and GS (cortex: 2.3-fold, *p* < 0.001; striatum: 1.4-fold, *p* = 0.06) (Fig. 3A, upper right panel) reflect an upregulation of physiological astrocytic functions to maintain homeostasis accompanied by an increased level of GFAP and VIM (Fig. 3A, upper left panel). In contrast to the unchanged levels of GLT-1 and GLAST in the cortex of MBP29-hα-syn mice, we detected a decreased striatal expression of both glutamate transporters (Fig. 3A, lower left panel). The latter findings imply the possibility of a reduced astrocytic capacity of glutamate reuptake within the striatum of MBP29-hα-syn mice. In addition, the increased expression levels of cortical GAP-43 levels were accompanied by an upregulation of GS. A similar pattern for both proteins, but less pronounced, was present in the striatum of MBP29-hα-syn mice. Since the expression levels of both glutamate transporter were solely reduced in the striatum of MBP29-hα-syn mice, we hypothesized that a regional heterogeneity of astrocytes is present at an early, pre-motor stage.

### Highly pure ATP1B2^+^ (ACSA2^+^) population by additional magnetic bead removal of O4^+^-cells

Our observation of differential expression of several astrocyte-associated transcripts and proteins in the striatum and cortex of MBP29-hα-syn mice prompted us to perform RNA sequencing of astrocytic populations. To this end, we designed a recent astrocyte isolation protocol using MACS via ATP1B2 sorting. Following cell isolation using ACSA2-MicroBeads, we still detected a higher number of O4^+^-cells (> 30%). We removed O4^+^-cells by an additional pre-sorting step labeling for O4^+^-cells. After FcR blocking, we incubated the cell suspension using anti-O4-Microbeads. Subsequently, we performed an anti-ACSA2-MicroBeads labeling step followed by the flow-through of the O4 population. To assess purity and viability after the two-step MACS approach, we performed flow cytometry (FC) analysis demonstrating a high yield for ACSA2^+^ cells (86.3%) in conjunction with a significant reduction of O4^+^ cells down to 5% without inducing cell death (Fig. 3B, left panel). For assessment of cell identity, complementary to the results of the flow cytometry analysis, we further applied the *enricher*-function of the clusterProfiler-package to merge the top 40 most highly expressed transcripts of the cortical and striatal datasets on a cell type annotation database [48]. We confirmed the identity of the isolated cell population as astrocytes (Fig. 3B, right panel). In addition, isolated cells express markers of neural stem cells and Bergmann glia known to share astrocytic transcriptional patterns (Table 3).

**Table 3:** Assessment of cell identity using clusterProfiler

Cell identity	Cell-type-specific markers
Astrocytes	GLUL, GPR37L1, ATP1A2, SLC1A2, CST3, S1PR1, BCAN, PLPP3, SPARCL1, SLC6A11, SLC6A1, PTN, MFGE8, APOE, TTYH1, HTRA1, GJA1, CPE, CLU, ALDOC, ATP1B2, SLC1A3, SCD2
Quiescent neural stem cell	ATP1A2, MFGE8, HTRA1, GJA1, CLU, ALDOC
Neural stem cell	MFGE8, APOE, HTRA1, CLU, ALDOC, SLC1A3
Bergmann glia	PLPP3, SPARCL1, SLC1A3
Macrophages	GLUL, CST3, S1PR1, MFGE8, APOE, HTRA1

### Transcriptional upregulation of pro-inflammatory transcripts accompanied by altered homeostasis-associated transcripts in the striatum of MBP29-hα-syn mice

Hypothesizing that the striatum presents a pro-inflammatory, hostile microenvironment, our next objective was to elucidate the specific transcriptional profile of isolated ACSA2^+^ astrocytes. Given the limited volume of the murine striatum and consequently the low RNA concentration, we employed a novel ultra-low amount sequencing technique. To ensure comprehensive transcriptomic data sets, we initially performed principal component analysis (PCA) to assess samples based on genotype clustering (Additional file 4: Fig. S3). A clear separation and clustering were present between both genotypes indicating a prominent divergence of transcriptional profiles and molecular signatures.

**Fig. 4:**
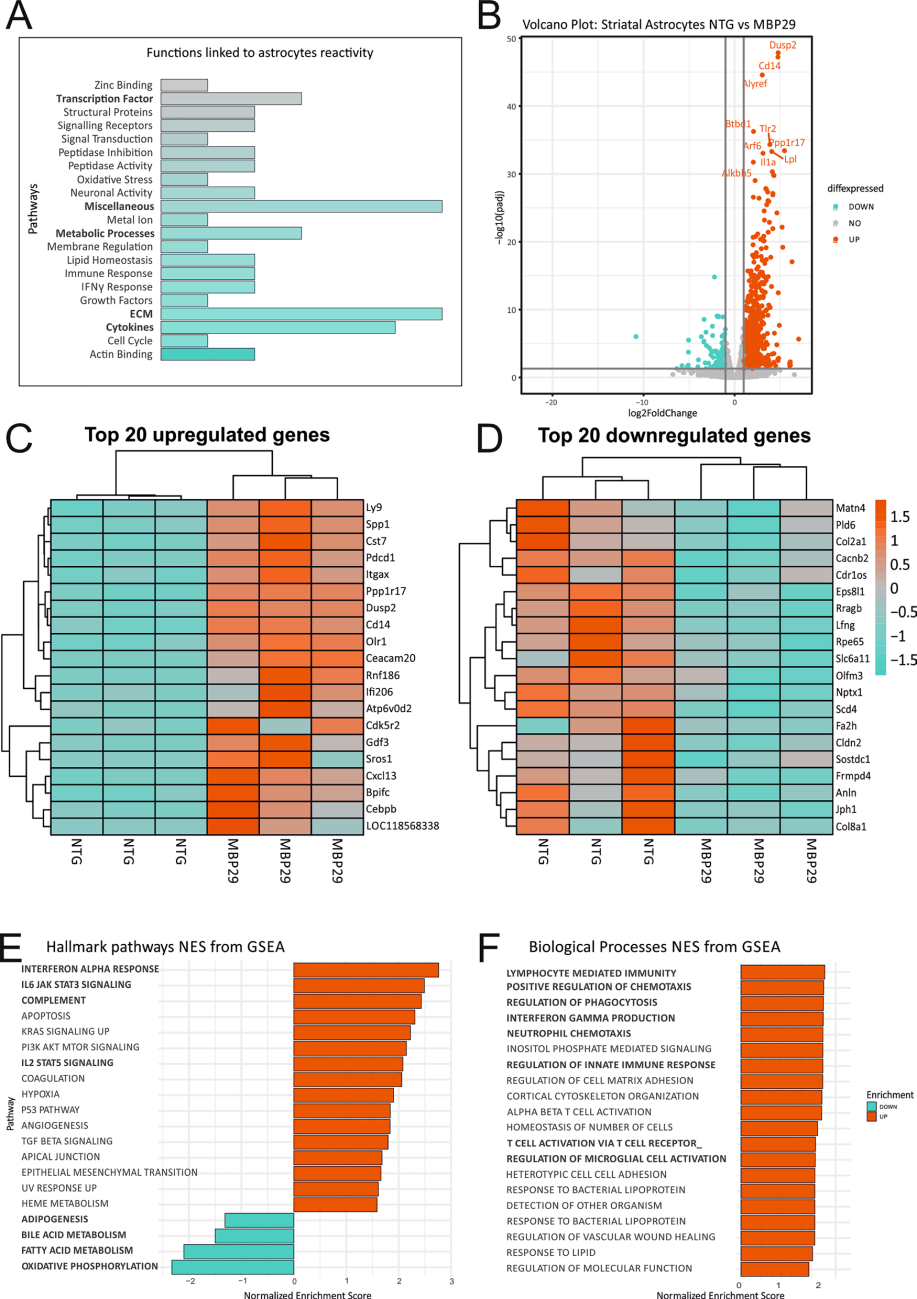
Transcriptome analysis of striatal astrocytes after MACS via ACSA2 of MBP29-hα-syn mice. **A** Mosaic plot of altered astrocytic functions in the striatum of MBP29-hα-syn mice based on existing dataset assessed by Zamanian et al. [16]. Astrocytes display a distinct upregulation of transcripts linked to astrocyte reactivity. Notably, there is a strong enrichment in cytokine activity, extracellular matrix (ECM) organization, metabolic processes and transcription factor activity, as well as not clearly classifiable functions (Miscellaneous). **B** Volcano plot of differentially expressed genes (DEGs). DEGs were analyzed comparing MBP29-hα-syn mice with non-transgenic littermates. Thresholds were set to log2foldchange > 2.0 and adj. *p* value < 0.05. Y-axis: negative decade logarithm of adj. *p* value. X-axis: log2foldchange of gene expression levels. In turquoise: significantly downregulated genes, in grey: genes without statistical significance, in red: significantly upregulated genes. Top hits were marked with official gene symbols by the HUGO Gene Nomenclature Committee (HGNC). **C**, **D** Heatmap of the 20 most highly up- and downregulated genes according to adjusted p-value. Gene expression was scaled by rows to visualize differences in gene expression between and within the genotypes using log2foldchange of gene expression. Striatal astrocytes demonstrate a profound upregulation of pro-inflammatory transcripts. Additionally, homeostatic functions, such as lipid metabolism, calcium transport, and neurotransmitter signaling are impaired in astrocytes on transcriptional level. **E**, **F** Functional gene set enrichment analysis of DEGs (FDR > 0.05). Pathways are ordered by normalized enrichment score (NES). Nomenclature of pathways is modified from official hallmark and biological processes pathways. Bold: Pathways associated with pro-inflammatory pathways and impaired homeostasis

Next, we assessed the presence of astrocyte-related transcripts and reactivity status to confirm that differentially expressed genes could be assigned to the striatal astrocyte population. We performed a comparative analysis by cross-referencing the striatum dataset with previously published reactivity-associated transcripts [16]. This approach provided us with a more in-depth insight into GO-term linked reactivity-associated astrocyte transcripts in transgenic mice (Fig. 4A, Table 4). Overall, striatal astrocytes of MBP29-hα-syn mice demonstrate upregulated transcripts linked to cytokine response, extracellular matrix organization, metabolic processes, transcription factors, and various processes not specifically assigned to distinct pathways (Additional file 8: Table S4).

**Table 4:** GO-Terms and associated transcripts differentially expressed in striatal astrocytes of MBP29-hα-syn mice

Pathway	Genes
Actin binding	ARPC1B, MSN
Cell cycle	CDKN1A
Cytokines	CCL2, CLCF1, CXCL2, IL6, SPP1
Extracellular matrix	CELA1, ECM1, ICAM1, IGFBP3, OLFML3, PVR
Growth factors	GDF15
Interferon-γ response	GBP3, IFI202B
Immune response	TLR4, CD14
Lipid homeostasis	CH25H, PLA2G4A
Membrane regulation	ANXA3
Metabolic processes	ASPG, OASL2, PDE3B
Metal ion	SLC39A14 ↓
Miscellaneous	AHNAK2, BTG3, IER3, LRRFIP1, SLC7A1, TSPAN4
Neuronal activity	GAP43, SYT4
Oxidative stress	GPX1
Peptidase activity/inhibition	LONRF1, USP18, SERPINE1, SERPING1
Signaling transduction/receptors	GADD45B, IL13RA1, TNFRSF12A
Structural proteins	GFAP, TUBB6
Transcription factors	BCL3, SBNO2, TGIF1
Zinc binding	RNF19B

Upon detailed analyses of the 20 most highly upregulated transcripts, the astrocytic pattern of the striatum relates to an immunological active signature via CD14, lymphocyte antigen 9 (Ly9), secreted phosphoprotein 1 (Spp1), cystatin F (Cst7), and programmed cell death 1 (PDCD1) associated pathways, the latter two being involved in amyloid angiopathies and multiple sclerosis [49, 50] (Fig. 4C). On the other hand, the 20 most highly downregulated genes rather indicate a diverse range of important biological processes and signaling pathways in striatal ACSA2^+^ astrocytes. In particular, transcripts identified are linked to metabolic pathways involved in lipid homeostasis of the CNS such as stearoyl-CoA desaturase (SCD-4) and fatty acid 2-hydroxylase (FA2H). As striatal astrocytes display downregulated transcripts involved in important cellular maintenance processes such as calcium homeostasis (matrilin 4, (MATN4) and calcium channel voltage-dependent subunit beta 2 (CACNB2)), and moreover neurotransmitter homeostasis such as gamma-aminobutyric acid (GABA) transport (solute carrier family 6 member 11 (SLC6A11)), and neuronal pentraxin 1 (NPTX1), these results highlight the impairment of essential astrocyte functions in the striatum of MBP29-hα-syn mice.

To obtain a more detailed overview of transcript enrichment in specific pathways, we applied functional gene set enrichment analysis (fgsea) on two different pathway sets: hallmark gene set for a generalized overview and KEGG for a more specific annotation. Our results were consistent with the initial analysis based on expression levels of the 20 differentially up-/downregulated genes, respectively (Fig. 4E, F). We detected a substantial enrichment of transcripts, particularly in interferon-α (NES = 2.6), complement (NES = 2.3), and interleukin-2/STAT5 signaling (NES = 1.97) confirming the pro-inflammatory signature of striatal astrocytes.

In conjunction with the pro-inflammatory qPCR findings in cells of striatal homogenates, it suggests that ACSA2^+^ astrocytes may drive neuroinflammatory processes within the striatum of MBP29-hα-syn mice. Moreover, striatal ACSA2^+^ astrocytes of transgenic mice show signs of an impaired lipid homeostasis and energy supply further implying astrocytic malfunctioning.

### Cortical astrocytes adopt an anti-inflammatory, secretory phenotype in MBP29-hα-syn mice

To characterize the transcriptional signature of cortical astrocytes, we performed an identical analysis as described for the striatum. The general count of differentially expressed transcripts based on previously published reactivity markers is much lower in cortical ACSA2^+^ astrocytes (Table 5). Furthermore, there is no association of transcripts linked to cytokine- or inflammatory responses in the isolated astrocytic population [16]. Interestingly, the sole transcript linked to immune response is lipocalin 2 (LCN2) showing a downregulation in cortical ACSA2^+^ astrocytes of MBP29-hα-syn mice.

**Table 5:** GO-Terms and associated transcripts differentially expressed in cortical astrocytes of MBP29-hα-syn mice

Pathway	Genes
Actin binding	FSCN1
Extracellular matrix	ADAMTS4, CELA1, OLFML3
Immune response	LCN2
Metabolic processes	ASPG, BCAT1, CTPS, PDE3B, UCK2
Miscellaneous	KLHDC8A, TMEM74
Mitochondria	MTHFD2
Neuronal activity	GAP43
Peptidase activity/inhibition	SERPINA3N
Signaling transduction/receptors	IL6RA
Structural proteins	GFAP
Zinc binding	NT5E

**Fig. 5:**
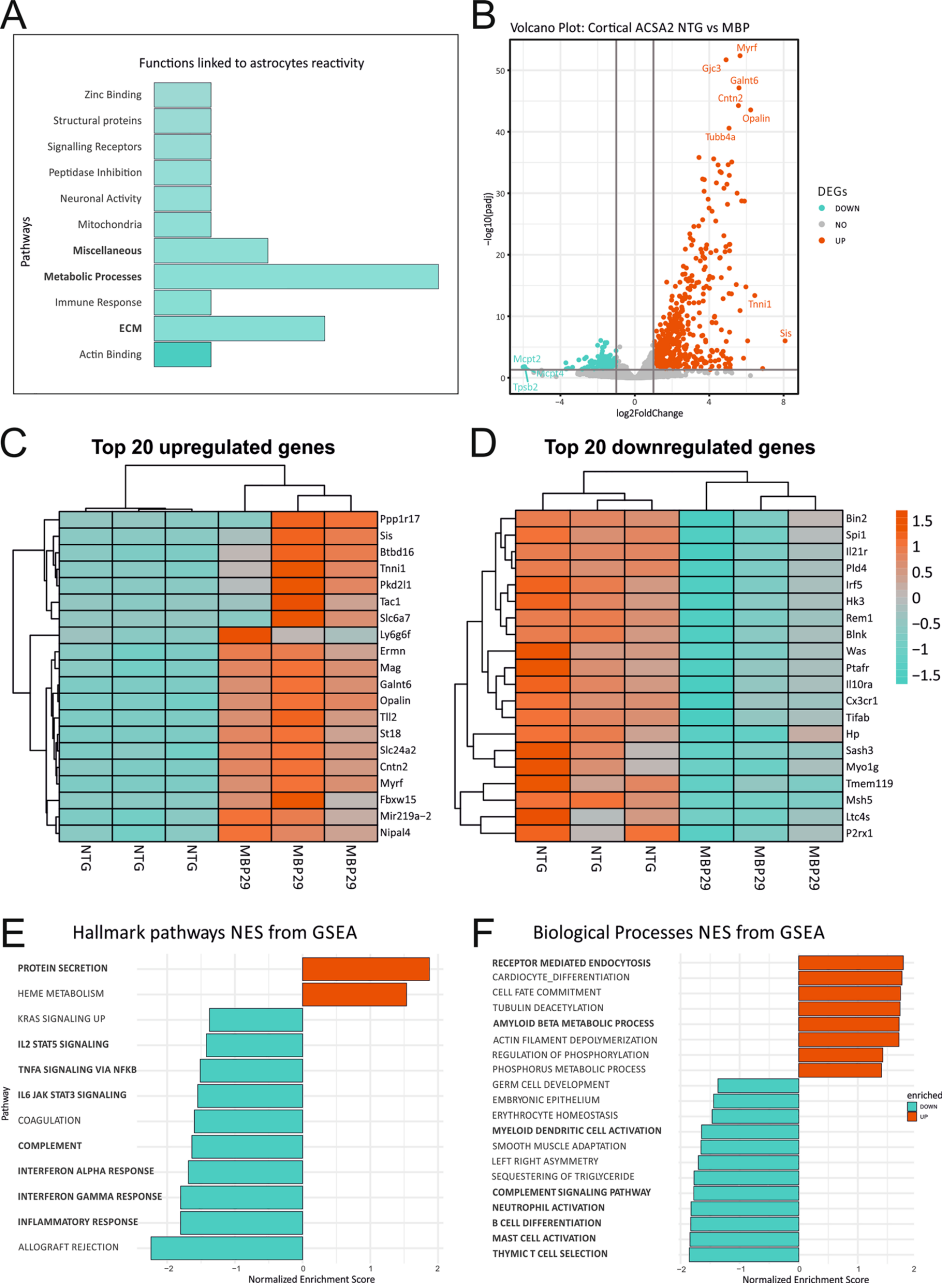
Transcriptome analysis of cortical astrocytes after MACS via ACSA2 of MBP29-hα-syn mice. **A** Mosaic plot of altered astrocytic functions in the cortex of MBP29-hα-syn mice based on existing dataset [16]. Reactivity profile of cortical astrocytes show less pronounced upregulation of reactivity-associated genes compared to striatal astrocytes. Primary processes affected refer to metabolic and ECM organization, as well as unclassified processes. **B** Volcano plot of DEGs analyzed comparing MBP29-hα-syn mice with non-transgenic litter mates. Thresholds were set to log2foldchange > 2.0 and adj. *p* value < 0.05. Y-axis: negative decade logarithm of adj. *p* value. X-axis: log2foldchange of gene expression levels. In turquoise: significantly downregulated genes, in grey: genes without statistical significance, in red: significantly upregulated genes. Transcripts with most significant adj. p-values and log2foldchange were visualized using official gene symbols by HGNC. **C**, **D** Heatmap of the 20 most highly up- and downregulated genes according to adjusted *p* value. Gene expression was scaled by rows to visualize differences in gene expression between and within the genotypes using log2foldchange of gene expression. Cortical astrocytes display significant upregulation of transcripts associated with oligodendrocyte development and myelination (ERMN, MAG, MYRF, OPALIN), ion and neurotransmitter homeostasis, and neuroprotection (SLC6A7, TLL2, SLCA24A2, SIS). **E**, **F** Functional gene set enrichment analysis of DEGs (FDR > 0.05). Pathways are ordered by normalized enrichment score (NES). Nomenclature of pathways is modified from official hallmark and biological processes pathways. **E** Transcripts enriched in protein secretion are upregulated in cortical astrocytes, whereas a significant downregulation of transcripts enriched in pro-inflammatory processes (IL-6/STAT3, complement, IFNγ, IFNα) was observed. **F** Upregulated transcripts demonstrate enrichment in receptor-mediated endocytosis and amyloid-β metabolism. Enrichment of downregulated transcripts mostly in immune system associated processes (complement, dendritic cell activation) confirms pattern already observed in hallmark analysis

The 20 most differentially expressed genes of cortical ACSA2^+^ astrocytes significantly differed from the striatal astrocytes of MBP29-hα-syn mice (Additional file 7: Table S3). Surprisingly, no transcripts involved in pro-inflammatory processes were upregulated in the cortex of transgenic mice. Moreover, essential transcripts for oligodendrocyte development and myelination were upregulated in cortical ACSA2^+^ astrocytes (ermin (ERMN), myelin-associated glycoprotein (MAG), oligodendrocytic myelin aranodal and inner loop protein (Opalin), and myelin regulatory factor (MYRF)). Besides the upregulation of oligodendrocyte-associated transcripts, we observed an elevation in the expression of genes associated with ion transport (solute carrier family 6 member 7 (SLC6A7), protein phosphatase 1 regulatory subunit 17 (PPP1R17), and tolloid like 2 (TLL2)), neurotransmitter homeostasis and neuroprotection (solute carrier family 24 member 2 (SLC24A2), contactin 2 (CNTN2), and platelet derived growth factor subunit B (SIS), Fig. 5B). These findings suggest that astrocytes isolated from the cortex of MBP29-hα-syn mice embrace a supportive role for surrounding cells.

Notably, in contrast to striatal astrocytes, cortical ACSA2^+^ astrocytes displayed a significant downregulation of several pro-inflammatory transcripts (bridging integrator 2 (BIN2), interleukin 21/10 receptor (IL21/10R), phospholipase D family member 4 (PLD4), interferon regulatory factor 5 (IRF5), and B Cell Linker (BLNK)). Additionally, we observed a downregulation of genes associated with lipid and energy homeostasis in cortical ACSA2^+^ astrocytes of MBP29-hα-syn mice (hexokinase 3 (HK3) and leukotriene C4 synthase (LTC4S)), as well as genes involved in actin-binding/regulation (RRAD and GEM Like GTPase 1 (REM1), Myosin IG (MYO1G), Fig. 5C, D).

For further characterization of cortical ACSA2^+^ astrocytes, we applied fgsea. Cortical astrocytes exhibit a rather secretory phenotype by upregulation of transcripts associated with protein secretion (Fig. 5E). In contrast to striatal ACSA2^+^ astrocytes, the overall inflammatory response was reduced in cortical astrocytes of MBP29-hα-syn mice, consistent with findings of the 20 most highly downregulated genes (Fig. 5E, F). Detailed analysis of biological processes revealed upregulated transcripts involved in receptor-mediated endocytosis and amyloid beta metabolic processes (Fig. 5F). The enrichment of downregulated pathways observed further supports the notion of particular immune system-associated processes being downregulated, as well as processes involved in sequestration of triglycerides suggested to be protective in lipotoxic conditions. In conjunction with findings obtained from striatal ACSA2^+^ astrocytes, it is evident that cortical and striatal ACSA2^+^ astrocytes exhibit a very distinct molecular pattern despite a similar degree of GFAP upregulation.

### Region-specific upregulation of pro-inflammatory transcripts in ACSA2^+^ astrocytes of MBP29-hα-syn mice

**Fig. 6:**
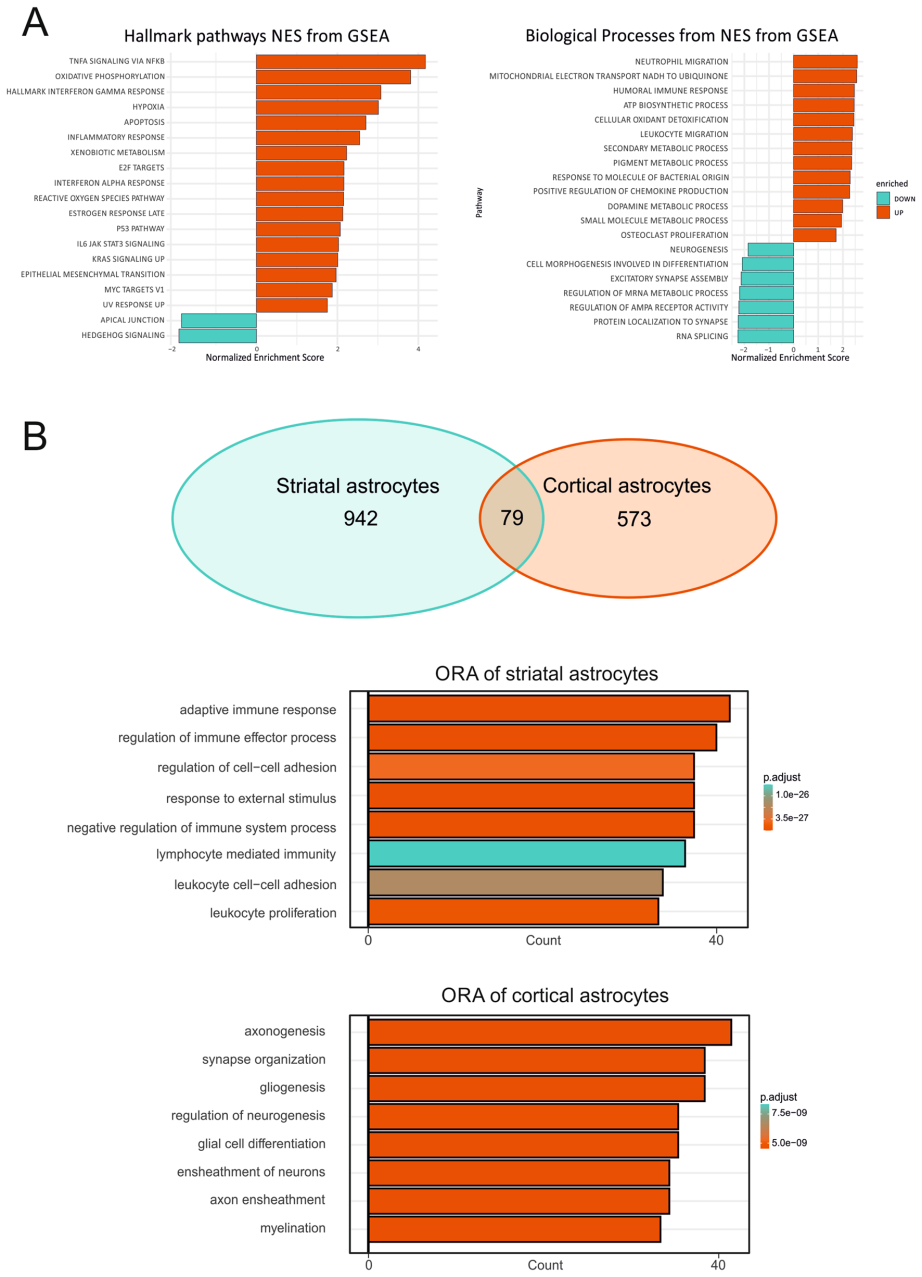
Comparison of cortex- and striatum-derived astrocytes from MBP29-hα-syn mice. **A** Functional gene set enrichment analysis based on DEGs. Striatal transcripts were compared to cortical transcripts and subsequently enriched in hallmark pathways and biological processes. Most significant enrichment of striatal transcripts were observed in pathways associated with a pro-inflammatory response and energy homeostasis, implying a more pronounced reactivity in striatum compared to the cortex. **B** Venn-diagram of overlapping genes of striatal and cortical transcripts derived from displaying 79 shared differentially expressed genes. Overrepresentation analysis of the non-overlapping genes (973 striatal, 573 cortical genes) displaying the top 8 overrepresented pathways sorted by the count of enriched transcript. Color code is based on the adj.* p*-value

To better delineate differences between striatal and cortical ACSA2^+^ astrocytes of MBP29-hα-syn mice, we conducted an analysis of differentially expressed genes (log2 fold change > 1.0, padj < 0.05) followed by gene set enrichment analysis (FDR < 0.05; Fig. 6A). In respect to the separate analyses of both brain regions, this approach confirmed that pro-inflammatory pathways (TNF signaling via Nfkb, interferon γ response, and inflammatory responses) are predominantly present in striatal ACSA2^+^ astrocytes. Merging both regional datasets demonstrates the clearly distinguishable transcriptomic profile of cortical and striatal astrocytes with a low total number of overlapping DEGs of 79 genes shown by Venn-diagram (Fig. 6B). Performing overrepresentation analysis (ORA) to display the non-overlapping genes (942 striatal vs 573 cortical transcripts) of both datasets, we confirmed that striatal astrocytes mainly exhibit an inflammation-associated molecular profile, regulate migration and cell adhesion, and responds to external stimuli (Fig. 6B, lower panel). Inversely, cortical astrocytes appear to be involved in supportive processes, such as axonogenesis, myelination, gliogenesis, and differentiation processes (Fig. 6B, upper panel). This ORA highlights the diversity of cortical and striatal astrocytes regarding their profile and potentially associated functions in the context of MSA-related pathology. Overall, we show that striatal astrocytes exhibit an increased immune response compared to cortical astrocytes.

### Oligodendroglia-associated transcripts are present within close proximity of astrocytic processes

**Fig. 7:**
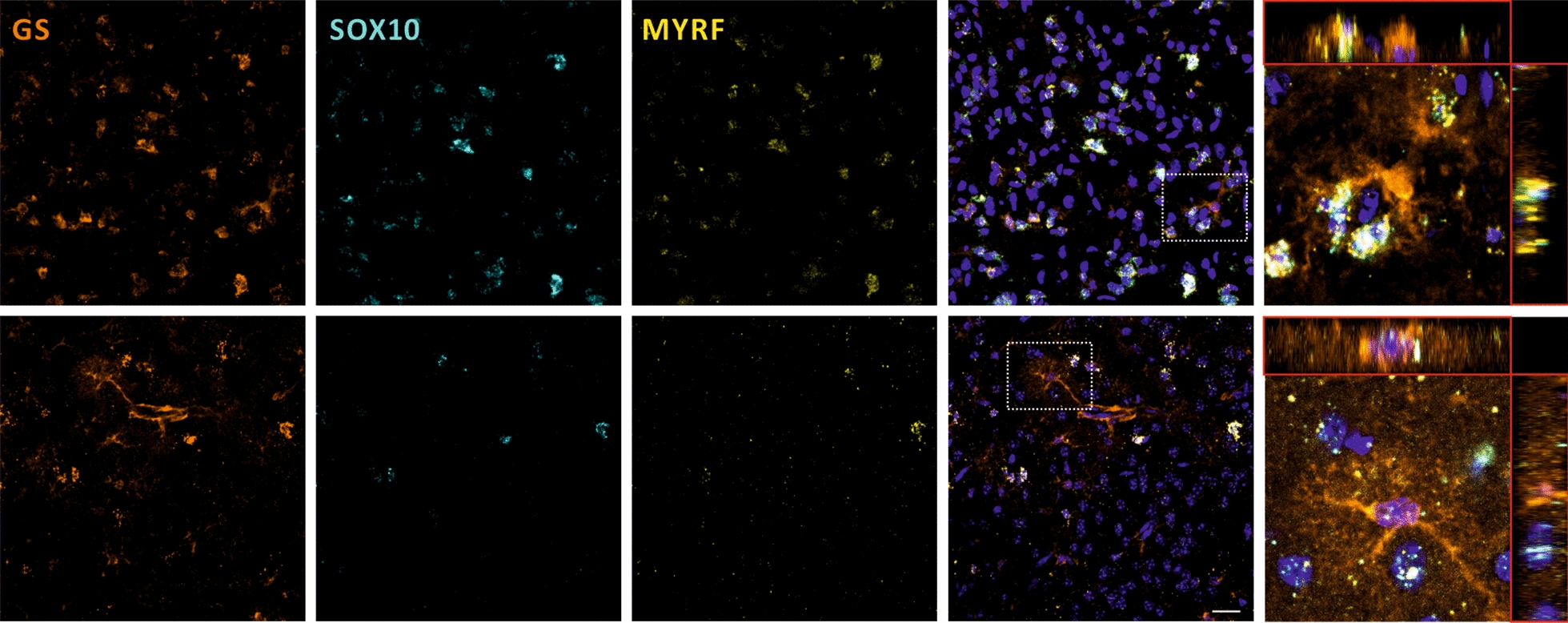
In-situ hybridization of oligodendrocyte-associated transcripts in the cortex of MBP29-hα-syn mice. RNAscope hybridization of oligodendroglial transcripts in astrocytes. GS was used as a pan-astrocyte marker (orange). Sox10 (cyan) and Myrf (yellow) were considered as oligodendrocyte markers covering characteristic of maturing and myelinating oligodendrocytes. Nuclei were counterstained using DAPI (blue). In MBP29-hα-syn mice, oligodendroglial co-label with astrocytic processes stained with GS, but are not unequivocally localized within the processes. Scale bar = 20 µm

As we observed a profound upregulation of pro-myelinogenic and oligodendroglial transcripts in cortical ACSA2^+^ astrocytes, we asked whether we are able to detect oligodendrocyte-associated transcripts in astrocytes in MBP29-hα-syn mice. To this end, we applied *in-situ* hybridization by RNAscope ISH Technology to detect oligodendroglial SRY-Box Transcription Factor 10 (SOX10) and MYRF transcripts in cortical GS^+^ astrocytes (Fig. 7). Indeed, we clearly detected *Sox10* and *Myrf * transcripts within GS^+^ astrocytic processes. Due to the lack of markers to visualize the entire network of astrocytic processes, we were not able to trace the transcripts to the respective nuclei of astrocytes. Nevertheless, the spatial proximity of astrocytic processes and oligodendroglial transcripts suggests that oligodendroglial transcripts may be located in the processes of astrocytes in the cortex of MBP29-hα-syn mice.

## Discussion

Reactive astrogliosis is a prominent feature in multiple neurodegenerative disorders [51]. Several studies taking advantage of microarray- and RNA sequencing technology and surface marker-based sorting of astrocytes emphasized region- and context-dependent diversity of astrocytes [16, 33–35, 52]. Here, we analyzed GFAP expression as a proxy for reactive astrogliosis in *post mortem* tissue of MSA-P patients in brain regions severely affected by disease pathologies such as the motor cortex, putamen, and substantia nigra. We detected an increase of GFAP^+^ cells suggesting reactive astrogliosis in all three regions. Notably, the putamen and the substantia nigra, most prominently affected in MSA-P, displayed a proportionally higher number of GFAP^+^ astrocytes compared to motor cortex. To extend the characterization of astrocytes, we analyzed expression of EAAT2, an important transporter for glutamate reuptake. A decrease of glutamate reuptake transporter expression has been linked to other neurodegenerative disorders, such as Alzheimer’s disease (AD) and amyotrophic lateral sclerosis (ALS) [53–56]. Quantifying GFAP^+^/EAAT2^+^ astrocytes hinted towards decreased numbers in MSA-P patients compared to controls and revealed altered distribution of EAAT2 in both brain regions.

Analyzing the expression of EAAT2 in the cortex and the putamen of MSA-P patients provided evidence of a lower number of GFAP^+^/EAAT2^+^ astrocytes in MSA-P patients. Notably, the distribution pattern of EAAT2 is altered in MSA-P patients in cortex and striatum, demonstrating a re-distribution from a diffuse to a nuclear pattern. This altered localization of EAAT2 was already described in vitro  in a model of AD upon exposure of astrocytes to Aβ_1-42_ [57]. The altered expression pattern results in a slower clearance time of excessive glutamate in the synaptic cleft and is therefore potentially linked to Aβ-related pathology. Based on this astrocytic phenotype in the human *post mortem* tissue, we further characterized astrocytes in MBP29-hα-syn mice. Mirroring the findings in human *post mortem* tissue, we also observed a substantial increase of GFAP^+^ cells in the cortex, the putamen and the substantia nigra. An enhanced astrocytic response was previously observed in grey compared to white matter regions of MSA-P patients and/or in MBP29-hα-syn mice [31]. The similarities in GFAP expression between MSA patients and the present transgenic MSA mouse model prompted us to a comprehensive analysis of astrocytes in distinct brain regions. Interestingly, a tendency of an increased total astrocyte number was present in the cortex of MBP29- hα-mice. Previous work showed an increased number of oligodendrocytes in the corpus callosum and preserved numbers of oligodendrocytes in the putamen of MBP29- hα-mice [32]. These findings are further supported by the observations of Ge and colleagues, hinting towards the presence of a glial progenitor population at the age between 3–4 weeks postnatally [58]. The increased levels of the reactivity-associated markers GFAP and VIM in the striatum suggest that these astrocytes may be much more responsive to α-syn pathology compared to other regions. This notion was accentuated by a decrease of GLT-1 and GLAST protein expression exclusively in the striatum potentially resulting in glutamate excess. The context-dependent expression levels of glutamate transporter were described in 6-hydroxydopamine-lesioned PD model [59]. Alternatively, the reduced levels of GLT-1 and GLAST in the striatum compared to the cortex hint towards the intrinsic heterogeneity between cortical and striatal astrocytes thus supporting the findings of a distinct profile of astrocytes in cortex and striatum of MBP29-hα-syn mice. Assessing the expression of additional homeostasis-associated astrocyte markers, we observed an upregulation of GS in the cortex while there was no significant change in the striatum. Lack of GS expression was already shown to impact neurodegenerative processes [60]. Furthermore, AQP-4 and GAP-43 protein levels were more increased in the cortex compared to the striatum suggesting that the capacity to maintain CNS homeostasis of striatal astrocytes is potentially impaired in MBP29-hα-syn mice.

To investigate the impact of MSA-related neuropathological processes on astrocytes on a cellular level, we developed an astrocyte isolation method with high purity modified from an existing surface marker-based sorting protocol using ATP1B2 as an unbiased astrocyte-specific surface marker [61] and subsequent astrocyte RNA sequencing. The bioinformatic analysis of the gene expression patterns revealed astrocyte identity in the isolated cell population but also indicated the pattern of neural stem cells. On the one hand, this enrichment might result from the shared expression patterns of astrocytes and neural stem cells [62]. On the other hand, since these mice are in a postnatal developmental period, this enrichment may be due to the presence of co-isolated neural stem cells residing in the subventricular zone close to the striatum [63]. Improving the isolation approach, we were able to achieve a high purity of ACSA2^+^ cells and high-quality outcome after bulk RNA sequencing. To evaluate astrocyte reactivity, we compared the astrocytic gene expression profile derived from the cortex and striatum MBP29-hα-syn mice to RNA-seq datasets previously published by Zamanian et al. [16] which induced reactive astrogliosis in wild-type mice using intraperitoneal lipopolysaccharide injections. Interestingly, the transcriptome of both astrocyte gene expression profiles overlapped with Zamanian et al., however, striatal astrocytes displayed an enrichment of these markers.

The most striking result to emerge by analyzing the cortex and the striatum, was a pro-inflammatory signature in striatal compared to cortical astrocytes. Elevated levels of pro-inflammatory cytokines were observed in the cerebrospinal fluid (CSF) of MSA patients [64]. Moreover, Valera and colleagues assessed pro-inflammatory cytokine levels in transgenic MSA mice [65]. In this study, the authors provide evidence of elevated CSF levels of IL-1β, IL-1ra, IL-13, IL-17, MIP-1α, and G-CSF using a cytokine proteomic assay matching the findings of the present transcriptomic findings. Additionally, region-dependent responses have been described in mouse models of Huntington’s disease [20, 66]. Striatal mouse astrocytes have been shown to respond context-specifically by G_i_-GPCR signaling in vivo with an upregulation of neuroinflammatory responses but whether the inflammatory response is toxic or protective remained unclear [66]. There is evidence that striatal astrocytes demonstrate altered transcriptomic profiles in inflammatory environment [67] associated with impaired homeostatic functions for calcium and glutamate signaling [68]. Distinct populations of astrocytes were identified in a HD mouse model exhibiting variable transcriptional phenotypes in the cortex reflecting a rather protective phenotype consistent with our findings [69, 70]. Another study characterizing astrocytes by single nuclei sorting in the prefrontal cortex, most severely affected in AD patients, revealed decreased homeostatic and increased levels of inflammatory transcripts [71]. In general, comparing astrocytes derived from AD patient’s *post mortem* brains, revealed non-homogenous astrocyte population including subpopulations capable of upregulating supportive processes as stress and survival response, but also subsets with metabolic stress and defective clearance [72]. Another remarkable result is that differentially expressed genes in striatal astrocytes are enriched in downregulated lipid-associated pathways and impaired energy homeostasis in the cortex of hereditary spastic paraplegia and AD patients [73, 74]. Taken together, a more inflammatory astrocytic response is present in CNS regions predominantly affected by the respective pathology.

Here, we assessed a pronounced pro-inflammatory state of astrocytes accompanied by impaired homeostatic processes via regional isolation and subsequent bulk RNA sequencing. The alteration of electrophysiological characteristics in neuroinflammatory conditions induced by brain abscesses has already been described in striatal astrocytes [75, 76]. Furthermore, an induction of neurotoxic astrocytes via release of microglial TNFα and IL1β has recently been shown [11]. In contrast, astrocytes are the major source of chemokines (CCL2, CXCL1 [77]) with corresponding receptors expressed by microglia potentially implicating a bi-directional communication. The upregulation of macrophage colony-stimulating factor (CSF-1) in astrocytes upon exposure to pro-inflammatory cytokines was observed which in turn suppressed microglial interferon- and MHC expression [78]. Another potential route of astrocyte-microglia communication is the microglial secretion of vascular endothelial growth factor β (VEGFB) for modulation of pathogenic characteristics of astrocytes [79]. In addition to the secretory component of astrocytes, components of the innate immune system play a pivotal role, such as toll-like receptors (TLR) and complement factors [80–82], which were observed to be upregulated in the striatum of MBP29-hα-syn mice. Especially, TLR4 is known as an essential receptor for astrocyte and microglia activation by aSyn [83] and could therefore play a crucial role in the crosstalk of both cell types. More detailed analysis of the complex interaction between microglia and astrocytes is necessary in future work to reveal potential mechanisms of cross-talk and maintenance of immune response in the CNS.

To our surprise, cortical astrocytes display an oligodendroglial and pro-myleinogenic expression pattern within the 20 most highly upregulated genes (ERMN, MAG, OPALIN, MYRF). Given the fact, that glutamate transporter expression is influenced by MSA pathology, we are not able to apply more specific isolation via the glutamate reuptake transporter GLAST (ACSA1). Nonetheless, we were able to determine high enrichment of astrocytes by bioinformatical enrichment algorithm. Moreover, we applied an RNA hybridization approach to confirm the presence of oligodendroglial transcription factors within or in proximity of astrocytic processes. A second limitation is due to the fact that the visualization of an entire astrocyte is difficult using commercially available antibodies against astrocyte markers, such as GFAP, ALDH1L1, S100β, and GS. Using RNAscope, we succeeded to visualize the presence of oligodendroglial transcripts in proximity of astrocytic processes counterstained with GFAP or GS. Combined with the enrichment of upregulated transcripts in cortical astrocytes involved in protein secretion, these results may potentially suggest that astrocytes support oligodendrocytes by energy supply, secreting trophic factors, and support of immune response as shown in other demyelinating diseases [84–87]. Furthermore, intercellular RNA transfer might serve as a possible mechanism for astrocytes to provide support to neighboring cells [88]. Future investigations are necessary to fully explore the specific potential of astrocytes to endogenously express distinct oligodendroglial transcripts thereby either promoting a change of cellular identity or supporting dysfunctional oligodendrocytes in the cortex of MBP29-hα-syn mice.

The present study provides a detailed region-specific characterization of the transcriptomic landscape of astrocytes in MSA. Our work contributes to a better understanding of astrocyte heterogeneity in distinct brain regions differentially affected by MSA-related neuropathology. The upregulation of reactivity-associated proteins in astrocytes, to certain limits, does not necessarily affect astrocytic capacity to support surrounding cells or the maintenance of CNS homeostasis in the affected regions. Moreover, despite reactive cortical astrogliosis, MBP29-hα-syn mice upregulate homeostasis related proteins such as AQP-4, GS, and GAP-43. In the striatum, severely affected by MSA pathology, we provided evidence for the presence of reactive astrocytes acquiring a more harmful phenotype by upregulation of pro-inflammatory transcripts and failing to maintain essential functions as energy and lipid homeostasis.

### Supplementary Information

**Additional file 1:** List of primers used for qPCR.

**Additional file 2: Fig. S1.** Overview of the region analyzed in human *post mortem* tissue. HE-staining of the prefrontal cortex (PFC), the putamen (PT), and the substantia nigra (SN) of frontally sectioned human brain.

**Additional file 3: Fig. S2.** Quantification of ALDH1L1 in the cortex the corpus callosum the striatum and the substantia nigra derived from MBP29-hα-syn mice and non-transgenic littermates. Left panel. ALDH1L1 was used as a pan-astrocyte marker to visualize all astrocytes independent of the state of reactivity. ALDH1L1^+^ (white) cell numbers (n = 6/group/region) were assessed and normalized to DAPI^+^ (blue) cells. Right panel. A slight increase of ALDH1L1^+^ cells was observed in all three regions not reaching statistical significance. In the cortex, a 1.5-fold increase of ALDH1L1^+^ cells were observed indicating a migration of astrocytes or new-born astrocytes in this region. CX = cortex, CC = corpus callosum, STR = striatum, SN = substantia nigra. Scale bar = 20 µm.

**Additional file 4: Fig. S3.** Principal component analysis (PCA) of isolated RNA from cortical and striatal tissue of MBP29-hα-syn mice. Clustering of MBP29 (orange) and NTG (green) samples after bulk RNA sequencing by PCA. Left panel. Variance within the cortical samples amount to 9% whereas the variance between the two genotypes is 86%. Right panel. Variance within the striatal samples amount to 13% whereas the variance between the two genotypes is 74%.

**Additional file 5: Fig. S4.** Expression levels of human aSyn in the cortex and the striatum of MBP29-hα-syn mice and non-transgenic littermates and assessment of myelin-associated protein expression levels. (A) Levels of aSyn were visualized using a human-aSyn specific antibody, demonstrating pronounced aSyn expression in MBP29-hα-syn mice compared to NTGs. Scale bar = 20 µm. (B) Quantification of myelination-associated proteins MBP and PLP demonstrates a 2-fold decrease of MBP (p = 0.0074) and a 3-fold decrease of PLP (p = 0.0053) expression in MBP29-hα-syn mice demonstrating a severe myelination deficit already at an age of 4 weeks.

**Additional file 6: Fig. S5.** Nuclear and diffuse distribution patterns of EAAT2 expressing astrocytes in MSA patients and controls. Analysis of expression patterns of EAAT2 reveals a nuclear and a diffuse expression pattern of EAAT2. Quantification demonstrates that there is a re-distribution of EAAT2 toward the astrocytic soma in the putamen of MSA patients compared to controls, however not reaching statistical significance (p = 0.057, Mann-Whitney-U test). Scale bar = 12.5 µm.

**Additional file 7**: Table of differentially expressed genes in the cortex of MBP29-hα-mice.

**Additional file 8**: Table of differentially expressed genes in the striatum of MBP29-hα-mice.
