# Supplementary material for: Distinct forebrain regions define a dichotomous astrocytic profile in multiple system atrophy
Source: Acta Neuropathol Commun. 2024 Jan 2;12:1. doi: 10.1186/s40478-023-01699-3 (PMC10759635; doi:10.1186/s40478-023-01699-3)
Supplement: Supplementary file 1 — Additional file 1: List of primers used for qPCR. [file 40478_2023_1699_MOESM1_ESM.docx]

**Supplementary Table S1** List of primers used for qPCR

| Gene | Primer pair (5’ – 3’) |
| --- | --- |
| GFAP | GCGTATAGACAGGAGGCAGA  TGGCCACATCCATCTCCAC |
| GLT-1 | TCTCCAGTTTAATCACAGGGTTG  ACCTCGTCGTTCTTCTTCCC |
| GLAST | TGCCAACAATATGCCCAAGC  GGCCTGACAACCCTGTGATT |
| Vimentin | AGACCAGAGATGGACAGGTGA  TTGCGCTCCTGAAAAACTGC |
| TNFα | GATGAGAAGTTCCCAAATGGCC  AGCCTTGTCCCTTGAAGAGA |
| IL1-β | TGACAGTGATGAGAATGACCTG  CCACGGGAAAGACACAGGTA |
| IL-6 | TGAGAAAAGAGTTGTGCAATGG  TCTCTCTGAAGGACTCTGGC |
| NFkB | GAGCAACCAAAACAGAGGGG  TTTGCAAAGCCAACCACCAT |
| C3 | AAGCATCAACACACCCAACA  CTTGAGCTCCATTCGTGACA |
| GUSB | AAGACATCGGGCTGGTGAC  TGTTGTCACCTTCACCTCCA |
| YWHAZ | AGACGGAAGGTGCTGAGAAA  TGACTGGTCCACAATTCCTTTC |

**Supplementary Table S2** Size of areas used for quantification of GFAP^+^ astrocytes in MSA-P patients and controls.

|  | Area PFC (µm^2^) | Area PPI (µm^2^) | Area SN (µm^2^) |
| --- | --- | --- | --- |
| MSA #1 | 1005910 | 1480800 | 2758350 |
| MSA #2 | 1724070 | 1969000 | 2328340 |
| MSA #3 | 1840290 | 1902110 | 2532120 |
| MSA #4 | 3203690 | 2885140 | 2616740 |
| Control #1 | 2776500 | 2604830 | 2482700 |
| Control #2 | 2069930 | 3718840 | 4409310 |
| Control #3 | 2338010 | 4220260 | 2871300 |
